# Legalizing Youth-Friendly Cannabis Edibles and Extracts and Adolescent Cannabis Use

**DOI:** 10.1001/jamanetworkopen.2025.5819

**Published:** 2025-04-18

**Authors:** Shweta Mital, Hai V. Nguyen

**Affiliations:** 1 College of Pharmacy, University of Manitoba, Winnipeg, Manitoba, Canada; 2 School of Pharmacy, Memorial University of Newfoundland, St John’s, Newfoundland and Labrador, Canada

## Abstract

**Importance:**

A year after dried cannabis was legalized across Canada in October 2018, Canadian provinces except Quebec legalized sales of cannabis edibles and extracts, including youth-friendly edibles such as cannabis chocolates, candies, and desserts and cannabis vaping products. Little is known about the association of this legalization with adolescent cannabis use.

**Objective:**

To investigate changes in adolescent cannabis use and cannabis harm perceptions associated with the legalization of youth-friendly cannabis edibles and extracts in Canada.

**Design, Setting, and Participants:**

This serial cross-sectional study analyzed data from students in grades 7 to 11 who participated in the nationally representative Canadian Student Tobacco Alcohol and Drugs Surveys in 2018 to 2019 and 2021 to 2022. A differences-in-differences design compared changes in outcomes in provinces that legalized cannabis edibles and extracts with changes in Quebec where youth-friendly cannabis edibles and cannabis vaping products were banned. Data were analyzed from June 2024 to January 2025.

**Exposure:**

Legalization of youth-friendly cannabis edibles and extracts across provinces in Canada (except Quebec) in October 2019.

**Main Outcomes and Measures:**

The primary outcomes were cannabis use in the past 12 months, past 12-month use of edible cannabis use, cannabis smoking, cannabis vaping, and co-use of alcohol and cannabis. Secondary outcomes were perception of harm from occasional and regular cannabis use.

**Results:**

In the study sample of 106 032 students in grades 7 to 11 (54 441 male [weighted percentage, 51.3%]), between 2018 to 2019 and 2021 to 2022, past 12-month cannabis use increased from 14.6% (6081 of 41 477 students) to 15.9% (6163 of 38 675 students) and use of edible cannabis increased from 7.9% (3268 of 41 373 students) to 9.5% (3678 of 38 556 students) in provinces that legalized youth-friendly cannabis edibles and extracts. Meanwhile, past 12-month cannabis use declined from 17.4% (2264 of 13 030 students) to 15.6% (1960 of 12 550 students) and use of edible cannabis declined from 7.3% (955 of 13 002 students) to 5.9% (739 of 12 533 students) in Quebec. Regression analyses indicated that the legalization was associated with a 3.8 percentage point (95% CI, 1.1 to 6.6 percentage points; *P* = .01) or 26% increase in past 12-month cannabis use and a 3.4 percentage point (95% CI, 1.9 to 4.9 percentage points; *P* = .001) or 43% increase in use of cannabis edibles among adolescents. While there was no statistically significant change in cannabis vaping associated with the legalization, cannabis smoking increased by 4.4 percentage points (95% CI, 1.8 to 7.0 percentage points; *P* = .004) or 34% and co-use of alcohol and cannabis increased by 2.4 percentage points (95% CI, 0.5 to 4.3 percentage points; *P* = .02) or 28%. The legalization was also associated with a lower perception of harm from occasional cannabis use.

**Conclusions and Relevance:**

In this serial cross-sectional study of adolescents in grades 7 to 11, legalization of cannabis edibles and extracts was associated with an increase not only in edible cannabis use and cannabis smoking, but also in the overall prevalence of cannabis use and co-use of alcohol and cannabis, highlighting the need for stricter policy measures to curb adolescents’ access to cannabis edibles and extracts and greater awareness among adolescents about harms of cannabis use.

## Key Points

**Question:**

What is the association of legalizing youth-friendly cannabis edibles and extracts with overall cannabis use prevalence, modes of cannabis use, and harm perceptions among adolescents in Canada?

**Findings:**

In this cross-sectional study of 106 032 adolescents, cannabis legalization was associated with a 26% increase in the overall prevalence of cannabis use, a 43% increase in edible cannabis use, a 34% increase in cannabis smoking, and a 28% increase in co-use of alcohol and cannabis. The legalization was also associated with lower perception of cannabis harms.

**Meaning:**

The increase in adolescents’ cannabis use associated with the legalization highlights the need for stricter policy measures to curb adolescents’ access to cannabis edibles and extracts and greater awareness among adolescents about harms of cannabis use.

## Teaser

This cross-sectional study analyzes changes in adolescent cannabis use and perceptions of cannabis harm associated with the legalization of youth-friendly cannabis edibles and extracts in Canada.

## Introduction

In October 2018, Canada legalized sale of dried or fresh cannabis and cannabis oil to individuals above the minimum legal age (set at 18 or 19 years across provinces).^1^ Subsequently, in October 2019, the sale of cannabis edibles and extracts was legalized across Canada, with the exception of Quebec. These products included items particularly appealing to adolescents, such as cannabis-infused chocolates, candies, and desserts and cannabis vaping products (referred to hereafter as youth-friendly cannabis edibles and extracts).^2^ Quebec banned the sale of youth-friendly cannabis edibles and vaping products to protect children and youths from accidental cannabis poisoning and to reduce the normalization of cannabis use among them. However, home production of these products was still allowed.^3^ The sale of certain non–youth-friendly edibles and extracts (such as dried cauliflower and dehydrated beets) was allowed in Quebec, but this only began in July 2022.^4^ Quebec’s government stated that the bans on youth-friendly cannabis edibles and extracts would protect children and youths from accidental cannabis poisoning and reduce normalization of cannabis use among youths.^5,6^ Three of the Atlantic provinces also restricted sales of cannabis vaping products, and bans on cannabis and hemp edibles have been made by health authorities in other jurisdictions in both Canada and the US.^7,8,9^

One of the intended goals of the cannabis legalization was to prevent adolescent cannabis use.^10^ However, leading up to this historic policy change, health professionals had strongly argued that the legalization would pose serious health risks for adolescents,^11^ highlighting the increased risk of psychiatric disorders and poor cognitive and adverse educational outcomes associated with cannabis use in adolescence.^12,13^ The legalization of youth-friendly cannabis edibles and extracts further heightened these concerns. Cannabis edibles are appealing to adolescents not only due to their taste (eg, chocolates, candy, desserts, and sodas) but also because they can be secretly consumed. A qualitative study of adolescents aged 15 to 17 years noted that those who are concerned about smoking or want to consume cannabis discreetly prefer edibles over smoking.^14^ Moreover, cannabis edibles carry a high risk of overdose due to the delayed appearance of psychoactive effects, difficulty in dosing, and differences in tetrahydrocannabinol (THC) metabolism.^15^ Meanwhile, the legalization of cannabis extracts can increase adolescent cannabis vaping. In the absence of well-enforced caps, cannabis vaping products often have higher THC potencies than other forms of cannabis, which can lead to both stronger psychoactive effects^16^ and increased respiratory risks.^17,18^ Cannabis vaping among adolescents is becoming increasingly popular^19^ and more than 75% of e-cigarette or vaping-associated lung injuries cases in the US have been associated with vaping THC products.^17^ Additionally, legalizing cannabis edibles and extracts may normalize cannabis use. Previous studies have shown that adolescents who use edible cannabis have a lower perception of cannabis risks than those who do not.^20,21^

There is also a question about whether legalization of youth-friendly cannabis edibles and extracts affects adolescent use of other substances such as alcohol. Literature suggests a positive association of cannabis use intensity with alcohol craving.^22^ If the legalization increases adolescent cannabis use, it could also encourage co-use of alcohol and cannabis.

Currently, there exists little evidence on the impact of legalizing youth-friendly cannabis edibles and extracts to inform these discussions. One study^23,24^ examined the association of dried and edible cannabis legalization with unintentional cannabis hospitalizations among children aged 0 to 9 years; it found that after dried cannabis was legalized across Canada, cannabis hospitalization rates increased similarly (nearly 3-fold) in all provinces. However, after cannabis edibles were legalized, hospitalization rates doubled in provinces that legalized edibles but did not change in Quebec.^23,24^

Our study has 3 objectives. Using data from a large survey of Canadian adolescents in grades 7 to 11 and difference-in-differences (DD) design, we first examined the association of legalization of youth-friendly cannabis edibles and extracts (referred to as legalization hereafter) with overall prevalence of adolescent cannabis use. Next, we studied the association of the legalization with modes of cannabis consumption (ie, cannabis edibles, cannabis smoking, and cannabis vaping) and the co-use of alcohol and cannabis. Finally, we assessed the association of the legalization with adolescents’ perception of harm from cannabis use. We hypothesized that the legalization was associated with increased cannabis use (via each mode), higher co-use of cannabis and alcohol, and lower perception of cannabis harms among adolescents.

## Methods

### Data Sources and Study Period

Because this cross-sectional study used deidentified publicly-available secondary data from existing national surveys, no informed consent or ethics approval was required based on Newfoundland and Labrador’s Health Research Ethics Board guidelines. The study followed the Strengthening the Reporting of Observational Studies in Epidemiology (STROBE) reporting guideline. We used individual-level data from the nationally representative Canadian Student Tobacco, Alcohol and Drugs Surveys (CSTADS). The CSTADS are repeated cross-sectional surveys that biennially interview more than 60 000 students in grades 7 to 12 across all 10 provinces,^25^ with an average response rate of 66%.^26^ These surveys are particularly well-suited for this study because they contain detailed information on cannabis and alcohol use among adolescents, including the mode of consuming cannabis. Data collection for these surveys starts in October to December of the survey year and finishes by May of the next year.

We used data from the 2018 to 2019 and 2021 to 2022 survey cycles of the CSTADS. The 2018 to 2019 cycle was conducted after dried cannabis was legalized but before cannabis edibles and extracts were legalized in Canada, while the 2021 to 2022 cycle was conducted after cannabis edibles and extracts were legalized.

### Study Sample

Our study sample included adolescents in grades 7 to 11 (aged approximately 12 to 17 years). We excluded students in grade 12 because these may comprise students aged 18 years or older (information on age was not available in CSTADS) who would be affected by the change in minimum legal age for cannabis from 18 to 21 years in Quebec that occurred shortly after the legalization in other provinces.

### Study Outcomes

Our primary outcomes of interest were past 12-month cannabis use (an indicator variable equal to 1 if the respondent had used cannabis in the 12 months preceding the survey and 0 otherwise) as well as modes of cannabis use in the past 12 months: cannabis edibles (1 if respondent used edible cannabis in past 12 months and 0 otherwise); cannabis smoking (1 if respondent smoked cannabis in past 12 months and 0 otherwise); and cannabis vaping (1 if respondent vaped cannabis in past 12 months and 0 otherwise). We also examined co-use of cannabis and alcohol (1 if respondent used both substances on the same occasion in the past 12 months and 0 otherwise). As secondary outcomes, we studied 4 measures of adolescents’ perception of cannabis harm, namely, harm from occasional and regular cannabis smoking and from occasional and regular use of other modes of cannabis. These were indicator variables equal to 1 if adolescents perceived moderate or great risk of harm and 0 if they perceived no risk or slight risk. The survey questions for these outcomes are provided in eTable 1 in Supplement 1.

### Statistical Analysis

We used the DD design^27,28,29,30,31^ to compare changes in outcomes in provinces that legalized youth-friendly cannabis edibles and extracts (ie, treated provinces) with changes in Quebec (where youth-friendly cannabis edibles and cannabis vaping products were banned). By using Quebec as a control province, the DD analyses allow us to account for the effects of confounding factors occurring around the time of the policy introduction. The key assumption underlying this approach was that, in the absence of the legalization, trends in outcomes in the provinces that legalized cannabis edibles and extracts would have been parallel to trends in Quebec.

The DD analyses were implemented using regression models where the covariate of interest was an indicator for the legalization (equal to 1 for respondents in all provinces except Quebec in the 2021 to 2022 survey cycle and 0 otherwise). The models controlled for respondents’ grade, sex/gender, and area of residence (rural or urban). They also included province indicators to control for all time-invariant characteristics of provinces and survey cycle indicators to control for secular changes in outcomes that were common to the treated provinces and Quebec across time.

Outcomes were modeled using linear probability regressions to produce unbiased estimates in fixed-effects analyses^32^ and for ease of interpreting the marginal effects.^33,34^ All estimates were weighted to account for survey design. Standard errors were clustered at the province level.^35^ Analyses were performed June 2024 to January 2025 with Stata 18 software (StataCorp).^36^ Tests were 2-sided, and a 5% significance level was used. Respondents with missing data on outcomes or covariates were excluded from the regression analyses (eTable 2 in Supplement 1 compares characteristics of included and excluded respondents).

We conducted several analyses to investigate the robustness of our results. First, we controlled for the provincial unemployment rate as a proxy for the economic climate in the province. Second, we accounted for differences in cannabis availability over time across provinces by controlling for the number of cannabis retail stores per 100 000 population^37^ during each survey cycle. Third, Quebec and Manitoba were the only provinces that banned home cultivation of cannabis.^38^ Home cultivation of cannabis has been shown to be associated with increased use of cannabis edibles but not cannabis vaping.^39^ Thus, to account for the possibility that our findings may be due to home cultivation bans, we reestimated the DD regressions comparing only Manitoba with Quebec. Fourth, 3 of the 4 Atlantic provinces (Newfoundland and Labrador, Nova Scotia and Prince Edward Island) restricted sales of cannabis vaping products.^40^ The Atlantic provinces were also relatively less affected by the COVID-19 pandemic (which occurred shortly after the legalization) than the rest of Canada.^41^ Thus, to rule out potential confounding from these factors, we excluded the Atlantic provinces from the set of treated provinces. Lastly, the DD analysis relies on the parallel trends assumption which requires that, in the absence of the legalization, the trends in outcomes in Quebec and the treated provinces would be the same. To test the validity of this assumption, we compared prelegalization trends in past 12-month cannabis use in Quebec vs the treated provinces using an event study design and data for the period 2014 to 2022. The event study analysis was not possible for the other primary outcomes because data for those outcomes were not collected prior to CSTADS 2018 to 2019 survey cycle.

## Results

### Descriptive Statistics

Our study sample included 106 032 students, including 80 452 students in treated provinces and 25 580 students in Quebec (Table 1). The sample was distributed almost equally across grades 7 to 11 and 54 441 students (weighted percentage, 51.3%) were male. The majority of the sample (89 200 students [weighted percentage, 84.1%]) resided in urban areas. The treated provinces were broadly similar to Quebec, except that the proportion of the sample residing in urban areas was lower (65 560 [weighted percentage, 81.5%] vs 23 922 of Y students [weighted percentage, 93.5%]) and the mean (SD) provincial unemployment rate was slightly higher in treated provinces than in Quebec (5.98 [1.07] vs 4.90 [0.29]).

**Table 1.** Characteristics of Study Sample^a^

Characteristic	Participants, No. (weighted %)		
	Full sample (N = 106 032)	Treated provinces (n = 80 452)	Quebec (n = 25 580)
Grade			
7	20 832 (19.6)	15 577 (19.4)	5286 (20.7)
8	20 704 (19.5)	15 534 (19.3)	5194 (20.3)
9	21 754 (20.5)	16 483 (20.5)	5274 (20.6)
10	21 414 (20.2)	16 373 (20.4)	5024 (19.6)
11	21 328 (20.1)	16 485 (20.5)	4803 (18.8)
Sex/gender^b^			
Male/man	54 441 (51.3)	41 462 (51.5)	12 958 (50.7)
Female/woman	51 591 (48.7)	38 990 (48.5)	12 622 (49.3)
Urban	89 200 (84.1)	65 560 (81.5)	23 922 (93.5)
Provincial unemployment rate, mean (SD)	5.74 (1.07)	5.98 (1.09)	4.90 (0.29)

^a^ Data are from the 2018 to 2019 and 2021 to 2022 Canadian Student Tobacco, Alcohol and Drugs Surveys. The sample includes all students in grades 7 to 11 who participated in these surveys. Treated provinces are provinces that legalized youth-friendly cannabis edibles and extracts and include Nova Scotia, Ontario, Prince Edward Island, New Brunswick, Newfoundland and Labrador, British Columbia, Manitoba, Alberta, and Saskatchewan.

^b^ Sex and gender were used interchangeably because the public use data files for the 2018 to 2019 Canadian Student Tobacco, Alcohol and Drugs Surveys cycle contained information on sex while those for the 2021 to 2022 Canadian Student Tobacco, Alcohol and Drugs Surveys cycle contained information on gender.

**Figure 1.**
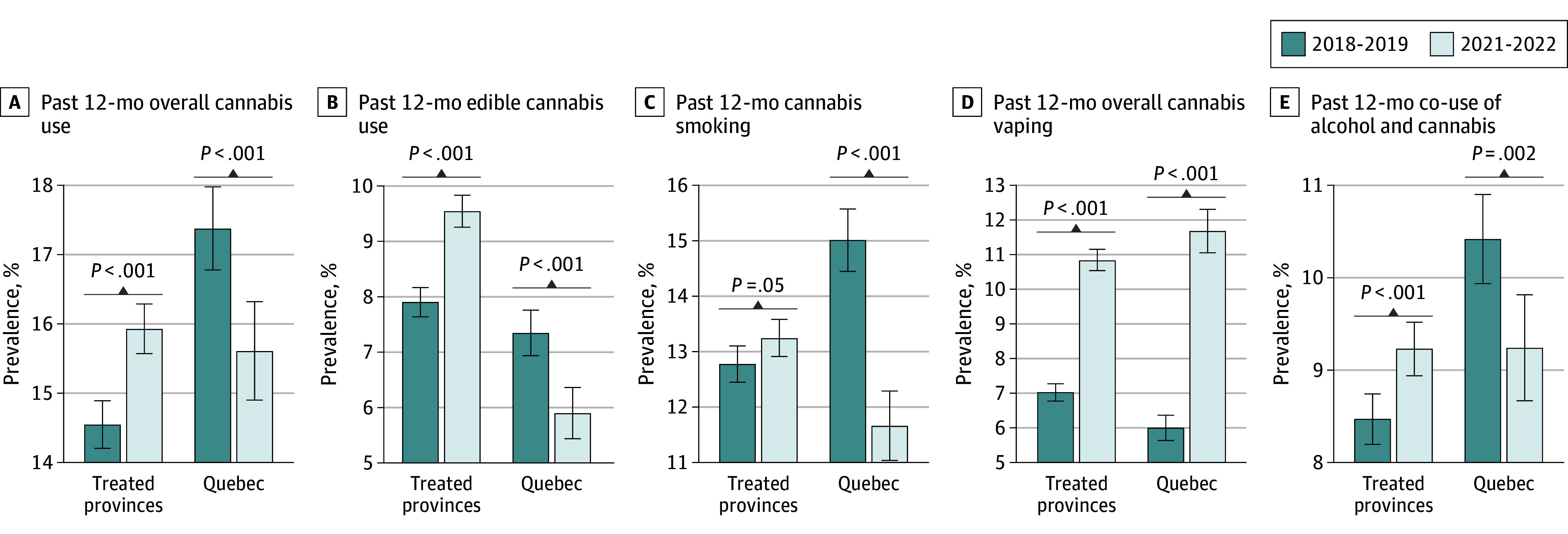
Changes in Past 12-Month Cannabis Use Prevalence, Edible Cannabis Use, Cannabis Smoking, Cannabis Vaping, and Co-Use of Cannabis and Alcohol in Provinces That Legalized Youth-Friendly Cannabis Data are from the 2018 to 2019 and 2021 to 2022 Canadian Student Tobacco, Alcohol and Drugs Surveys. The sample includes all students in grades 7 to 11. Treated provinces are provinces that legalized youth-friendly cannabis edibles and extracts and include Nova Scotia, Ontario, Prince Edward Island, New Brunswick, Newfoundland and Labrador, British Columbia, Manitoba, Alberta, and Saskatchewan. Whiskers indicate 95% CIs.

Figure 1 shows changes in outcomes in the treated provinces and Quebec before (2018-2019) and after (2021-2022) the legalization. Past 12-month cannabis use increased (from 14.6% [6081 of 41 477 students] to 15.9% [6163 of 38 675 students]; *P* < .001) after youth-friendly cannabis edibles and extracts became legal in the treated provinces while it declined in Quebec (from 17.4% [2264 of 13 030 students] to 15.6% [1960 of 12 550 students]; *P* < .001). Use of edible cannabis was similar between the treated provinces and Quebec before the legalization. After the legalization, it increased in the treated provinces (7.9% [3268 of 41 373 students] to 9.5% [3678 of 38 556 students]; *P* < .001) but declined in Quebec (7.3% [955 of 13 002 students] to 5.9% [739 of 12 533 students]; *P* < .001). A similar pattern was observed for cannabis smoking and co-use of alcohol and cannabis. Meanwhile, cannabis vaping increased in all provinces, but the increase in the treated provinces was slightly smaller than in Quebec.

After youth-friendly cannabis edibles and extracts were legalized, fewer adolescents in the treated provinces believed that occasional cannabis smoking or other modes of cannabis use posed moderate or great harm; no such decline was observed in Quebec (eFigure 1 in Supplement 1). The proportion of adolescents who believed in moderate or great harm from regular cannabis smoking or other modes of cannabis use declined similarly in both the treated provinces and Quebec.

### Regression Results

Table 2 displays the regression results. The top panel shows results from the base case analysis. The results indicate that the legalization was associated with a 3.8 percentage point increase (95% CI, 1.1 to 6.6 percentage points; *P* = .01) in past 12-month cannabis use. Edible cannabis use increased by 3.4 percentage points (95% CI, 1.9 to 4.9 percentage points; *P* = .001) in the treated provinces after the legalization compared with the corresponding change in Quebec. Compared with the prelegalization prevalence of 7.9% in the treated provinces, this increase represents a 43% increase in edible cannabis use. The legalization was also associated with a 4.4 percentage point (95% CI, 1.8 to 7.0 percentage point; *P* = .004) increase in cannabis smoking, representing a 34% increase relative to the prelegalization prevalence of 12.8% in the treated provinces. There was no statistically significant difference in the increase in cannabis vaping between the treated provinces and Quebec. Meanwhile, we found a 2.4 percentage point (95% CI, 0.5 to 4.3 percentage point; *P* = .02) increase in co-use of cannabis and alcohol associated with the legalization. Compared with prelegalization prevalence of co-use of 8.5% in the treated provinces, this estimate represents a 28% increase in co-use of alcohol and cannabis.

**Table 2.** Changes in Overall Cannabis Use, Edible Cannabis Use, Cannabis Smoking, Cannabis Vaping, and Co-Use of Alcohol and Cannabis Associated With Legalization of Youth-Friendly Cannabis Edibles and Extracts^a^

Outcome	Percentage point change associated with legalization (95% CI)	*P* value
Primary analysis		
Changes in overall cannabis use and modes of using cannabis		
Past 12-mo cannabis use (n = 106 032)	3.8 (1.1 to 6.6)	.01
Past 12-mo edible cannabis use (n = 105 464)	3.4 (1.9 to 4.9)	.001
Past 12-mo cannabis smoking (n = 105 756)	4.4 (1.8 to 7.0)	.004
Past 12-mo cannabis vaping (n = 105 422)	−1.6 (−3.7 to 0.6)	.14
Past 12-mo co-use of cannabis and alcohol (n = 103 103)	2.4 (0.5 to 4.3)	.02
Sensitivity analyses		
Control for provincial unemployment rate		
Past 12-mo cannabis use (n = 106 032)	3.2 (0.7 to 5.8)	.02
Past 12-mo edible cannabis use (n = 105 464)	2.9 (1.2 to 4.7)	.005
Past 12-mo cannabis smoking (n = 105 756)	2.7 (0.0 to 5.3)	.05
Past 12-mo cannabis vaping (n = 105 422)	−0.8 (−3.0 to 1.4)	.42
Past 12-mo co-use of cannabis and alcohol (n = 103 103)	1.8 (0.0 to 3.6)	.05
Control for number of cannabis stores		
Past 12-mo cannabis use (n = 106 032)	3.5 (−2.6 to 9.6)	.22
Past 12-mo edible cannabis use (n = 105 464)	3.9 (0.4 to 7.3)	.03
Past 12-mo cannabis smoking (n = 105 756)	4.5 (−1.1 to 10.0)	.10
Past 12-mo cannabis vaping (n = 105 422)	−1.4 (−7.1 to 4.2)	.58
Past 12-mo co-use of cannabis and alcohol (n = 103 103)	2.0 (−2.2 to 6.2)	.31
Only Manitoba as treated province		
Past 12-mo cannabis use (n = 31 274)	3.7 (0.3 to 7.1)	.05
Past 12-mo edible cannabis use (n = 31 206)	2.1 (0.0 to 4.2)	.05
Past 12-mo cannabis smoking (n = 31 246)	2.6 (1.2 to 4.0)	.03
Past 12-mo cannabis vaping (n = 31 189)	−1.7 (−3.3 to −0.1)	.16
Past 12-mo co-use of cannabis and alcohol (n = 30 658)	1.5 (0.3 to2.7)	.04
Excluding Atlantic provinces as treated provinces		
Past 12-mo cannabis use (n = 73 747)	3.9 (0.7 to 7.1)	.03
Past 12-mo edible cannabis use (n = 73 406)	3.4 (1.6 to 5.2)	.005
Past 12-mo cannabis smoking (n = 73 572)	4.4 (1.4 to 7.5)	.01
Past 12-mo cannabis vaping (n = 73 378)	−1.7 (−4.3 to 1.0)	.16
Past 12-mo co-use of cannabis and alcohol (n = 71 831)	2.5 (0.2 to 4.7)	.04

^a^ Data are from the 2018 to 2019 and 2021 to 2022 Canadian Student Tobacco, Alcohol and Drugs Surveys. All models are estimated using ordinary least squares regressions and control for individual-level factors: age, sex/gender (male; female is the excluded category), urban residence (rural is the excluded category) as well as province and year fixed effects. Standard errors clustered at the province level.

**Figure 2.**
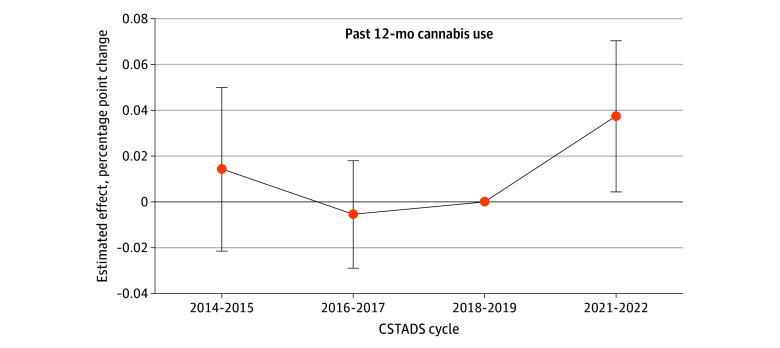
Event Study Analysis of Past 12-Month Cannabis Use Data are from the 2014 to 2015 through 2021 to 2022 Canadian Student Tobacco, Alcohol and Drugs Surveys (CSTADS). The sample includes all students in grades 7 to 11. Shown are estimated effects (in percentage point changes) from difference-in-differences regressions in which a single policy indicator variable is replaced by a series of event time indicators for CSTADS cycles before and after youth-friendly cannabis edibles and extracts were legalized. Error bars represent 95% CIs. The first CSTADS cycle prelegalization (2018-2019) is the reference time period.

The sensitivity analyses showed that our results were robust to the inclusion of provincial unemployment rate as a control (Table 2). When we controlled for the number of cannabis retail stores in operation in each province, the coefficient estimates for changes in overall cannabis use prevalence, cannabis smoking, and co-use of alcohol and cannabis were no longer statistically significant. However, the magnitudes of these estimates were very similar to the main analysis. We obtained similar results to the base case analysis when we compared Quebec with only Manitoba and when we excluded the Atlantic provinces, suggesting that our results were unlikely to be affected by home cultivation bans, restrictions on cannabis vaping products in the Atlantic provinces, or the COVID-19 pandemic. The graphical trend comparison as well as the event study revealed no systematic differences in trends in past 12-month cannabis use between Quebec and the treated provinces, confirming the validity of the parallel trends assumption (eFigure 2 in Supplement 1 and Figure 2, respectively).

Table 3 examines the association of the legalization with adolescents’ perception of harms from cannabis use. We found that the legalization was associated with a 5.6 percentage point (95% CI, 4.9 to 6.4 percentage point; *P* < .001) and 5.2 percentage point (95% CI, 4.1 to 6.3 percentage point; *P* < .001) lower likelihood that adolescents perceived moderate or great harm from occasional cannabis smoking and occasional use of other modes of cannabis, respectively. There was no change in perception of harm from regular cannabis smoking and regular use of other modes of cannabis associated with the legalization.

**Table 3.** Changes in Harm Perception From Occasional and Regular Cannabis Use Associated With Legalization of Youth-Friendly Cannabis Edibles and Extracts^a^

Outcome	Percentage point change associated with legalization (95% CI)	*P* value
Occasional cannabis smoking (n = 93 865)	−5.6 (−6.4 to −4.9)	<.001
Regular cannabis smoking (n = 93 273)	−0.6 (−1.4 to 0.1)	.09
Occasional use of other modes of cannabis (n = 89 050)	−5.2 (−6.3 to 4.1)	<.001
Regular use of other modes of cannabis (n = 89 497)	−0.3 (−0.8 to 0.3)	.32

^a^ Data are from the 2018 to 2019 and 2021 to 2022 Canadian Student Tobacco, Alcohol and Drugs Surveys. The outcome variables are binary variables equal to 1 if respondents perceive a moderate or great risk of harm and 0 if they perceive no risk or slight risk of harm. All models are estimated using ordinary least squares regressions and control for individual-level factors: age, sex/gender (male/man; female/woman is the excluded category), urban residence (rural is the excluded category) as well as province and year fixed effects. Standard errors clustered at the province level.

## Discussion

To our knowledge, this cross-sectional study provides the first evidence on the association of legalizing youth-friendly cannabis edibles and extracts with adolescents’ prevalence and modes of cannabis use, co-use of alcohol and cannabis, and perception of harm from cannabis use. We found that the legalization was associated with an increase not only in edible cannabis use and cannabis smoking, but also the overall prevalence of cannabis use and co-use of alcohol and cannabis among adolescents. There was also a reduction in perception of harm from occasional cannabis use associated with the legalization.

Previous studies have found no significant change in adolescent cannabis use prevalence following the legalization of dried cannabis.^21,42,43^ Our finding of increased overall cannabis use and reduced harm perception associated with legalizing cannabis edibles and extracts therefore suggests that availability of these products—often perceived as safer alternatives to combustible forms of cannabis^44^—may have had a stronger influence on adolescents’ attitudes and the social acceptability of cannabis use compared with the legalization of dried cannabis.

It is not surprising that the use of cannabis edibles and cannabis vaping increased in provinces that legalized cannabis edibles and extracts. The small increase in cannabis smoking in these provinces likely occurred because adolescents who have used multiple modes of cannabis often prefer cannabis smoking.^45^ Yet, our findings were also attributable, in part, to the decline in cannabis smoking and edible cannabis use and the large increase in cannabis vaping seen in Quebec despite its ban on cannabis extracts. Lower perceived risks of vaping compared with smoking,^44^ the growing popularity of adolescent vaping, and the prompt effects and greater predictability of vaping highs relative to cannabis edibles may explain why adolescents in Quebec sourced cannabis vaping products from family and friends, legal sources in other provinces, and online.^46^

The federal Canadian law requires uniform color, child-resistant plain packaging, and labeling for all cannabis products with restrictions on brand name and logo.^47^ A THC symbol and details of THC and cannabidiol content are also required on the package.^47^ However, media reports indicate that packaging of available illicit edible cannabis products often resembles other treats.^48,49^ Adolescents may also not be aware of the presence of THC when offered a cannabis edible or be able to accurately estimate its level in a product.^49^ Educating adolescents about cannabis edibles before they try and then perhaps become regular users of these products will, therefore, be important.

Evidence suggests that those who use edibles often also engage in other modes of cannabis use^50^ and that youths who want to conceal use may be more likely to use edibles.^14^ Our finding of increased edible cannabis use, alongside increases in cannabis smoking and overall prevalence of cannabis use associated with legalization of cannabis edibles and extracts, suggests a potential positive association of edible cannabis use with other modes of cannabis use. Future studies could further explore these associations to better understand the interplay between different modes of cannabis use.

### Limitations

Our study has a number of limitations. First, the data on study outcomes were self-reported and may be subject to desirability bias. Further, the survey only included youths present at school; those absent from school, who may be at increased risk of cannabis use, were not captured in the surveys. However, assuming the effects of such desirability bias and sampling bias were static over time, they would be cancelled out in the DD analysis. Second, the surveys collected information on only past-year use of different types of cannabis products. Our study was therefore unable to shed light on the association of the policy with frequency of use of each product. Third, political changes occurred in Quebec in 2018 with the incumbent government advocating for stricter cannabis policies. These political changes could have potentially affected public sentiments toward cannabis use, although the changes occurred prior to the 2018 to 2019 survey cycle and would therefore be accounted for by province-specific fixed effects in the DD analyses. Further, in January 2020, Quebec increased the minimum legal age for cannabis from 18 to 21 years. While this policy change occurred around the same time as legalization of cannabis edibles and extracts, it is unlikely to explain our findings. Students in grades 7 to 11 were younger than 18 years both before and after the policy change, and the sources from which they obtained cannabis remained quite similar. In fact, the proportion of adolescents obtaining cannabis from family and friends increased between 2018 and 2021.^25^ Fourth, data on study outcomes were available for only 1 survey cycle after the legalization. To the extent that users may take time to adjust sourcing of different cannabis products as well as use patterns, future studies could examine the long-term association of the legalization with adolescent cannabis use behaviors as more data become available.

## Conclusions

This study found that the legalization of youth-friendly cannabis edibles and extracts was associated with an increase in not only edible cannabis use and cannabis smoking, but also in overall cannabis use prevalence among adolescents. There was evidence of increase in co-use of alcohol and cannabis associated with the legalization. Adolescents’ perception of harm from occasional cannabis use also declined. These findings highlight the need for additional policy measures to prevent adolescents’ access to cannabis edibles and extracts and to raise greater awareness of the harms of cannabis use during adolescence.

## Notes

Supplement 1. **eTable 1.** Survey Questions for Outcome Variables **eTable 2.** Baseline Characteristics Between Respondents Included in Each Regression Sample and Those Excluded due to Missing Values on Outcomes, Covariates or Both **eFigure 1.** Changes in Harm Perception From Cannabis Smoking and Other Modes of Cannabis Use, Provinces That Legalized Youth-Friendly Cannabis Edibles and Extracts vs Quebec **eFigure 2.** Trends in Past 12 Month Cannabis Use, Treated Provinces vs Quebec, 2014/15 – 2021/22

Supplement 2.
Data Sharing Statement
