# Supplementary material for: Legalizing Youth-Friendly Cannabis Edibles and Extracts and Adolescent Cannabis Use
Source: JAMA Netw Open. 2025 Apr 18;8(4):e255819. doi: 10.1001/jamanetworkopen.2025.5819 (PMC12008758; doi:10.1001/jamanetworkopen.2025.5819)
Supplement: Supplement 1. — eTable 1. Survey Questions for Outcome Variables eTable 2. Baseline Characteristics Between Respondents Included in Each Regression Sample and Those Excluded due to Missing Values on Outcomes, Covariates or Both eFigure 1. Changes in Harm Perception From Cannabis Smoking and Other Modes of Cannabis Use, Provinces That Legalized Youth-Friendly Cannabis Edibles and Extracts vs Quebec eFigure 2. Trends in Past 12 Month Cannabis Use, Treated Provinces vs Quebec, 2014/15 – 2021/22 [file jamanetwopen-e255819-s001.pdf]

Supplemental Online Content

Mital S, Nguyen HV. Legalizing youth-friendly cannabis edibles and extracts and adolescent cannabis use. *JAMA Network Open*. 2025;8(4):e255819. doi:10.1001/jamanetworkopen.2025.5819

eTable 1. Survey Questions for Outcome Variables
eTable 2. Baseline Characteristics Between Respondents Included in Each Regression Sample and Those Excluded due to Missing Values on Outcomes, Covariates or Both
eFigure 1. Changes in Harm Perception From Cannabis Smoking and Other Modes of Cannabis Use, Provinces That Legalized Youth-Friendly Cannabis Edibles and Extracts vs Quebec
eFigure 2. Trends in Past 12 Month Cannabis Use, Treated Provinces vs Quebec, 2014/15 – 2021/22

This supplemental material has been provided by the authors to give readers additional information about their work.

© 2025 Mital S et al. *JAMA Network Open*.

eTable 1. Survey Questions for Outcome Variables

Outcome	Variable definition	Based on survey question
Past 12-month cannabis use	Indicator (1 if a respondent reported using cannabis during past 12 months, 0 otherwise)	"*In the last 12 months, how often did you use marijuana or cannabis? - I have not done this in the last 12 months/ Less than once a month / Once a month / 2 or 3 times a month / Once a week / 2 or 3 times a week / 4 to 6 times a week / Every day*"
Past 12-month edible cannabis use	Indicator (1 if a respondent who had used cannabis in past 12 months reported using edible cannabis in the past 12 months, 0 otherwise)	"*Indicate whether you have used marijuana or cannabis (a joint, pot, weed, hash, or hash oil) in the following ways: Eaten it in food such as brownies, cakes, cookies or candy or drank it in tea, cola, alcohol, or other drinks - No, I have never done this / Yes, I have done this in the last 12 months / Yes, I have done this, but not in the last 12 months"*
Past 12-month cannabis smoking	Indicator (1 if a respondent who had used cannabis in past 12 months reported smoking cannabis in the past 12 months, 0 otherwise)	"*Indicate whether you have used marijuana or cannabis (a joint, pot, weed, hash, or hash oil) in the following ways: Smoked a joint, bong, pipe or blunt - No, I have never done this / Yes, I have done this in the last 12 months / Yes, I have done this, but not in the last 12 months"*
Past 12-month cannabis vaping	Indicator (1 if a respondent who had used cannabis in past 12 months reported vaping cannabis in the past 12 months, 0 otherwise)	"*Indicate whether you have used marijuana or cannabis (a joint, pot, weed, hash, or hash oil) in the following ways: Vaporized it (vape) - No, I have never done this / Yes, I have done this in the last 12 months / Yes, I have done this, but not in the last 12 months"*
Past 12-month co-use of cannabis and alcohol	Indicator (1 if a respondent used both alcohol and cannabis on the same occasion in the past 12 months, 0 otherwise)	*"In the last 12 months, how often did you have alcohol AND marijuana or cannabis on the same occasion? (e.g., at a party, in the same evening, etc.) - I have never had alcohol AND cannabis on one occasion / I have not done this in the last 12 months / Less than once a month / Once a month / 2 to 3 times a month / Once a week / 2 to 5 times a week / Daily or almost daily"*
Moderate/Great harm from occasional cannabis smoking	Indicator (1 if a respondent believed there was moderate or great risk of harm from occasional cannabis smoking, 0 if respondent believed there was no risk or slight risk)	*"How much do you think people risk harming themselves when they (Smoke marijuana or cannabis once in a while)?: No risk /Slight risk / Moderate risk / Great risk"*

© 2025 Mital S et al. *JAMA Network Open*.

Moderate/Great harm from regular cannabis smoking	Indicator (1 if a respondent believed there was moderate or great risk of harm from regular cannabis smoking, 0 if respondent believed there was no risk or slight risk)	*"How much do you think people risk harming themselves when they (Smoke marijuana or cannabis on a regular basis)?: No risk /Slight risk / Moderate risk / Great risk"*
Moderate/Great harm from occasional other modes of cannabis use	Indicator (1 if a respondent believed there was moderate or great risk of harm from occasional use of other modes of cannabis, 0 if respondent believed there was no risk or slight risk)	*"How much do you think people risk harming themselves when they (Other than smoking it, use marijuana or cannabis once in a while)?: No risk /Slight risk / Moderate risk / Great risk"*
Moderate/Great harm from regular other modes of cannabis use	Indicator (1 if a respondent believed there was moderate or great risk of harm from regular use of other modes of cannabis, 0 if respondent believed there was no risk or slight risk)	*"How much do you think people risk harming themselves when they (Other than smoking it, use marijuana or cannabis on a regular basis)?: No risk /Slight risk / Moderate risk / Great risk"*

Note: Survey questions are from CSTADS 2018/2019 and 2021/2022.

© 2025 Mital S et al. *JAMA Network Open*.

eTable 2. Baseline Characteristics Between Respondents Included in Each Regression Sample and Those Excluded due to Missing Values on Outcomes, Covariates or Both

Regression sample for outcome ⬇	Proportions in included sample	Proportions in excluded sample
Past 12-month cannabis use	Grade 7: 22.3%<br>Grade 8: 21.9%<br>Grade 9: 20.3%<br>Grade 10: 19.2%<br>Grade 11: 16.3%<br>Urban: 79.2%	Grade 7: 21.3%<br>Grade 8: 21.3%<br>Grade 9: 21.5%<br>Grade 10: 18.6%<br>Grade 11: 17.3%<br>Urban: 83.0%
Past 12-month edible cannabis use	Grade 7: 22.4%<br>Grade 8: 22.0%<br>Grade 9: 20.2%<br>Grade 10: 19.1%<br>Grade 11: 16.3%<br>Urban: 79.3%	Grade 7: 20.3%<br>Grade 8: 20.6%<br>Grade 9: 21.8%<br>Grade 10: 19.5%<br>Grade 11: 17.8%<br>Urban: 81.9%
Past 12-month cannabis smoking	Grade 7: 22.4%<br>Grade 8: 21.9%<br>Grade 9: 20.3%<br>Grade 10: 19.1%<br>Grade 11: 16.3%<br>Urban: 79.3%	Grade 7: 20.6%<br>Grade 8: 21.1%<br>Grade 9: 21.4%<br>Grade 10: 19.0%<br>Grade 11: 17.8%<br>Urban: 82.5%
Past 12-month cannabis vaping	Grade 7: 22.4%<br>Grade 8: 22.0%<br>Grade 9: 20.3%<br>Grade 10: 19.1%<br>Grade 11: 16.3%<br>Urban: 79.3%	Grade 7: 20.4%<br>Grade 8: 20.6%<br>Grade 9: 21.4%<br>Grade 10: 19.7%<br>Grade 11: 17.9%<br>Urban: 81.8%
Past 12-month co-use of cannabis and alcohol	Grade 7: 22.3%<br>Grade 8: 21.9%<br>Grade 9: 20.3%<br>Grade 10: 19.2%<br>Grade 11: 16.3%<br>Urban: 79.3%	Grade 7: 22.4%<br>Grade 8: 21.5%<br>Grade 9: 20.4%<br>Grade 10: 18.5%<br>Grade 11: 17.3%<br>Urban: 80.8%

Note: We do not compare sex/gender as this variable accounted for majority of the missing values in the excluded sample.

© 2025 Mital S et al. *JAMA Network Open.*

eFigure 1. Changes in Harm Perception From Cannabis Smoking and Other Modes of Cannabis Use, Provinces That Legalized Youth-Friendly Cannabis Edibles and Extracts vs Quebec

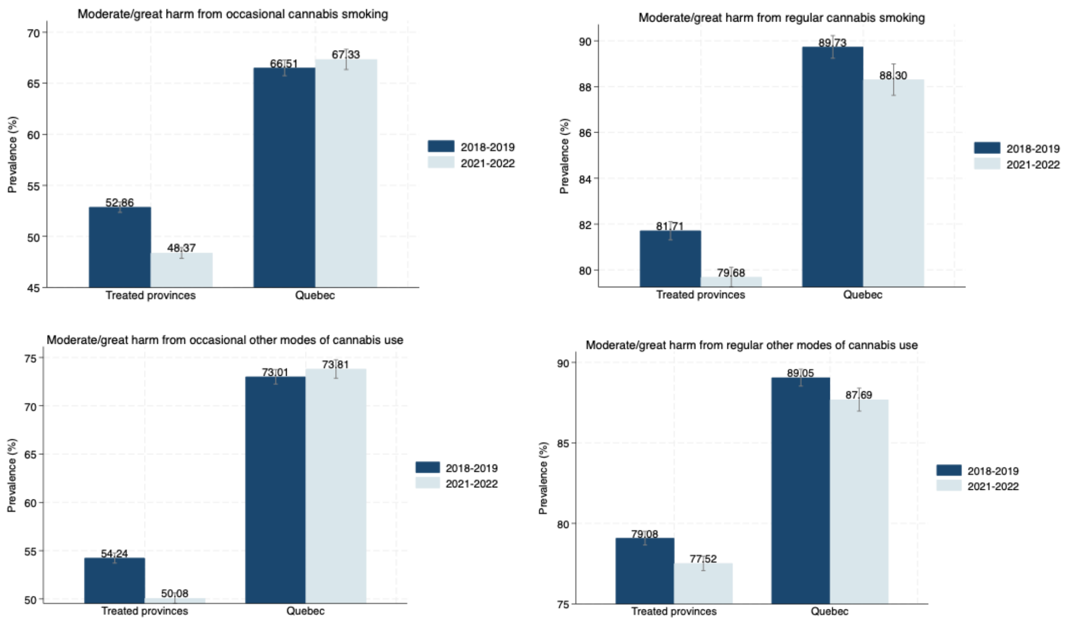

Note: Data are from CSTADS 2018/19 and CSTADS 2021/22. Sample includes all students in grades 7-11. Treated provinces include Nova Scotia, Ontario, Prince Edward Island, New Brunswick, Newfoundland and Labrador, British Columbia, Manitoba, Alberta and Saskatchewan.

© 2025 Mital S et al. *JAMA Network Open*.

eFigure 2. Trends in Past 12 Month Cannabis Use, Treated Provinces vs Quebec, 2014/15 – 2021/22

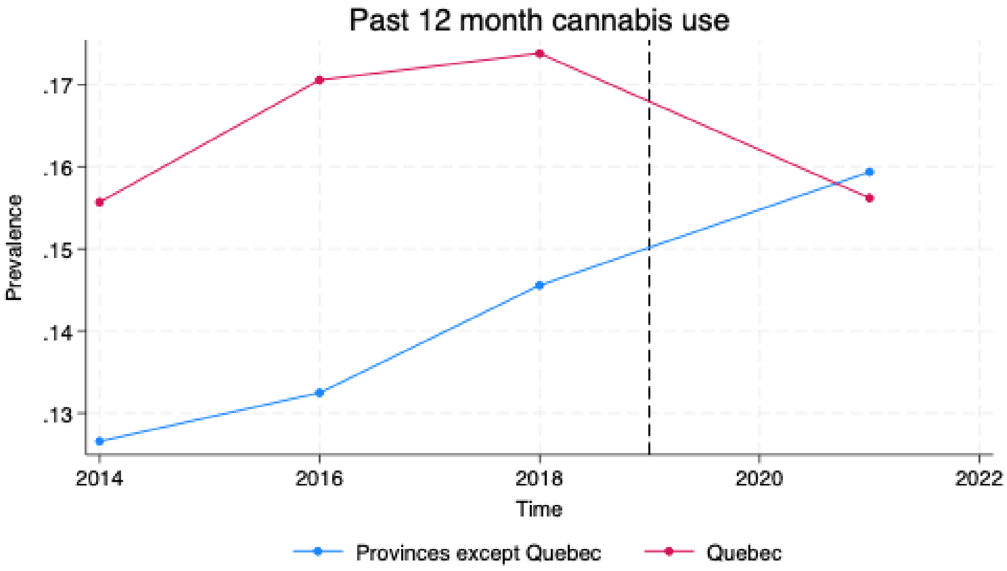

Note: Data are from CSTADS 2014/15 – 2021/22 for students in Grades 7 to 11.

© 2025 Mital S et al. *JAMA Network Open*.
