# Supplementary material for: Legalizing Youth-Friendly Cannabis Edibles and Extracts and Adolescent Cannabis Use
Source: JAMA Netw Open. 2025 Apr 18;8(4):e255819. doi: 10.1001/jamanetworkopen.2025.5819 (PMC12008758; doi:10.1001/jamanetworkopen.2025.5819)
Supplement: Supplement 2. — Data Sharing Statement [file jamanetwopen-e255819-s002.pdf]

Data Sharing Statement

Mital. Legalizing Youth-Friendly Cannabis Edibles and Extracts and Adolescent Cannabis Use. *JAMA Netw Open*. Published April 18, 2025. doi:10.1001/jamanetworkopen.2025.5819

Data
Data available: No

Additional Information
Explanation for why data not available: This study used publicly available data
